# 67 Patient Self-grading of Itch within Hypertrophic Scar Does Not Correlate to Mast Cell Concentration

**DOI:** 10.1093/jbcr/irad045.041

**Published:** 2023-05-15

**Authors:** Esteban Molina, Taryn Travis, Melissa McLawhorn, Jeffrey Shupp, Bonnie Carney

**Affiliations:** Georgetown University School of Medicine, Firefighter's Burn and Surgical Research Laboratory, MedStar Health Research Institute, Washington, DC; MedStar Washington Hospital Center, Washington, DC; Firefighters' Burn and Surgical Research Laboratory, MedStar Washington Hospital Center, Washington, DC; Firefighters' Burn and Surgical Research Laboratory, MedStar Health Research Institute, Georgetown University School of Medicine, Washington, DC; Firefighters' Burn and Surgical Research Laboratory, Medstar Health Research Institute, Georgetown University Medical Center, Washington, DC; Georgetown University School of Medicine, Firefighter's Burn and Surgical Research Laboratory, MedStar Health Research Institute, Washington, DC; MedStar Washington Hospital Center, Washington, DC; Firefighters' Burn and Surgical Research Laboratory, MedStar Washington Hospital Center, Washington, DC; Firefighters' Burn and Surgical Research Laboratory, MedStar Health Research Institute, Georgetown University School of Medicine, Washington, DC; Firefighters' Burn and Surgical Research Laboratory, Medstar Health Research Institute, Georgetown University Medical Center, Washington, DC; Georgetown University School of Medicine, Firefighter's Burn and Surgical Research Laboratory, MedStar Health Research Institute, Washington, DC; MedStar Washington Hospital Center, Washington, DC; Firefighters' Burn and Surgical Research Laboratory, MedStar Washington Hospital Center, Washington, DC; Firefighters' Burn and Surgical Research Laboratory, MedStar Health Research Institute, Georgetown University School of Medicine, Washington, DC; Firefighters' Burn and Surgical Research Laboratory, Medstar Health Research Institute, Georgetown University Medical Center, Washington, DC; Georgetown University School of Medicine, Firefighter's Burn and Surgical Research Laboratory, MedStar Health Research Institute, Washington, DC; MedStar Washington Hospital Center, Washington, DC; Firefighters' Burn and Surgical Research Laboratory, MedStar Washington Hospital Center, Washington, DC; Firefighters' Burn and Surgical Research Laboratory, MedStar Health Research Institute, Georgetown University School of Medicine, Washington, DC; Firefighters' Burn and Surgical Research Laboratory, Medstar Health Research Institute, Georgetown University Medical Center, Washington, DC; Georgetown University School of Medicine, Firefighter's Burn and Surgical Research Laboratory, MedStar Health Research Institute, Washington, DC; MedStar Washington Hospital Center, Washington, DC; Firefighters' Burn and Surgical Research Laboratory, MedStar Washington Hospital Center, Washington, DC; Firefighters' Burn and Surgical Research Laboratory, MedStar Health Research Institute, Georgetown University School of Medicine, Washington, DC; Firefighters' Burn and Surgical Research Laboratory, Medstar Health Research Institute, Georgetown University Medical Center, Washington, DC

## Abstract

**Introduction:**

Pruritus is a common and disturbing symptom among patients with hypertrophic scars (HTSs) that contributes to morbidity after burn injury. It can impact quality of life through sleep disturbance, limitation of daily activities, and psychosocial impairment. The pathophysiology and mechanisms behind HTS itch remain unclear but HTSs have previously been reported to have higher substance P (SP) nerve fiber density, greater SP levels, and an increased number of mast cells. Itch may be due to multiple contributing factors, and is commonly treated with various drugs with different targets including anti-histamines to target mast cells. We hypothesized that patients with self-reported high itch in HTS would have a higher concentration of mast cells compared to HTSs reported to have low itch. Here, we further investigate the involvement of mast cells in HTS itch.

**Methods:**

Patients with post-burn HTS were enrolled into an observational clinical trial. Patient and observer scar assessment scales (POSAS) were used to assess itch, among other parameters. Punch biopsies were collected from regions of HTS and normal skin (NS). Biopsies were embedded in paraffin, sectioned, and stained with Giemsa stain to identify mast cells. Patients were stratified by POSAS-itch scores into high ( >5, n=6 patients) and low (< 5, n=5 patients) and mast cell numbers were compared between the two groups using a Student’s t-test.

**Results:**

The high itch group was 83.3% female while the low itch group was 40.0% female. Other patient demographics did not significantly differ between the high itch group and the low itch group, including age (47±7.25 vs 48±7.07 years), Fitzpatrick skin type (5/6 type V vs 2/5 type V), and age of scar (8.4±2.16 vs 25.5±9.95 months). On histologic examination, the high itch group had an average of 16.25±24.1 mast cells per high powered field vs 5.70±3.6 mast cells in the low itch group (p=0.336). Regardless of itch score, HTS had an average of 11.45±18.1 mast cells vs 5.7±5.4 mast cells in normal skin (p=0.334).

**Conclusions:**

HTSs self-graded as having high itch did not have a significantly different number of mast cells present in tissue punch biopsies compared to biopsies of HTSs self-graded as low itch. Mechanisms behind scar itch remain unclear, but are likely due to other factors that are more contributory than the presence of mast cells. Further investigation of scar itch is warranted due to the burden associated with this distressful symptom.

**Applicability of Research to Practice:**

This research shows that mast cells may not be as contributory to itch symptomatology as previously thought, which may help explain why traditional antihistaminergics are often ineffective for HTS itch. Elucidating other pathways, such as those initiated by laser treatment which has been shown to decrease itch, may provide more opportunity to characterize potential targets to effectively treat HTS itch.

